# Audit Cycle to Assess Appropriate Usage of FP10 Prescriptions in Local Community Teams Against NICE-BNF Guidelines and Trust Policy

**DOI:** 10.1192/bjo.2026.11654

**Published:** 2026-06-30

**Authors:** Chalani Munasinghe, Joanne Henson

**Affiliations:** Mersey Care NHS Trust, Prescot, United Kingdom

## Abstract

**Aims:**

To assess the appropriate use of FP-10 prescriptions against the NICE-BNF and Trust Guidelines.

**Methods:**

This was a retrospective audit, and reaudit to include all FP-10 prescriptions written for the patients coming under the Community mental health teams in Halton (that is Assessment and HTT, Recovery Team, EIP, LLAMS and CAMHS).The sample was identified from Appendix 3 data sheets,RiOnotes, Clinical documents.The data were extracted from Data from existing databases, electronic systems and case notes.The total size of the population was 23 for the initial audit and 97 for the re-audit.The sample time period for the initial audit was 01/01/2022 till 25/07/2022 and reaudit was from 07/07/2024 to 07/08/2024. The sample selection was all consecutive FP 10 prescriptions used during the time period.

**Results:**

Initial audit had 97% overall compliance level but the criteria on completing appendix 3 for the completed FP-10s and scanning or documenting the FP-10s to patient records were met with a moderate compliance level of 83% and 87% respectively.

Action plan following the audit was to include a column in Appendix 3 to identify NHS numbers of the patients given FP-10s and to insert an information sheet to FP-10 bundle with guidance on recording details.

In both audits 100% compliance was met for storing the FP-10s and managing them securely and Prescription writing guidelines were followed according to NICE- BNF standards. This was not a prescribing audit and the doses and appropriateness of medication were not audited

Following the action plan in the re-audit the compliance level was met at 99% with the above-mentioned criteria improving compliance to 100% and 97% respectively.

**Conclusion:**

In both audits the FP-10s were given to prescribe psychotropics only and were written according to NICE-BNF guidance.

In both audits the FP-10s were stored and managed according to the local trust guidance.

The recording of FP-10 details improved following the simple intervention of including a prompt sheet with the prescriptions.

A secondary outcome was that the procedure for management of FP10s in the community has been reviewed and rewritten.

